# Predictors of One-Year Mortality in Hospitalized Patients with Splenic Infarction: Survival Analysis of a Retrospective Cohort in Taiwan

**DOI:** 10.7150/ijms.130149

**Published:** 2026-04-23

**Authors:** Jin-Wei Lin, Yu Kuo, Mei-Jy Jeng, Chien-Chien Tzeng, Chorng-Kuang How, Teh-Fu Hsu, David Hung-Tsang Yen, Hsien-Hao Huang

**Affiliations:** 1 Department of Emergency Medicine, Taipei Veterans General Hospital, Taipei, Taiwan.; 2 Institute of Emergency and Critical Care Medicine, National Yang Ming Chiao Tung University, Taipei, Taiwan.; 3 School of Medicine, College of Medicine, National Yang Ming Chiao Tung University, Taipei, Taiwan.; 4 Department of Radiology, Taipei Veterans General Hospital, Taipei, Taiwan.; 5 Department of Nuclear Medicine, Taipei Veterans General Hospital, Taipei, Taiwan.; 6 Department of Pediatrics, Taipei Veterans General Hospital, Taipei, Taiwan.; 7 Nursing Department, Taipei Veterans General Hospital, Taipei, Taiwan.; 8 Institute of Clinical Nursing, National Yang Ming Chiao Tung University, Taipei, Taiwan.; 9 Kinmen Hospital, Ministry of Health and Welfare, Kinmen, Taiwan.

**Keywords:** splenic infarction, survival analysis, mortality, risk factors, hospitalization

## Abstract

**Background:**

Splenic infarction, an uncommon disease, has been demonstrated to be associated with substantial short-term mortality. Given the limited evidence on long-term mortality, this investigation examined the clinical and radiological characteristics of splenic infarction, its long-term prognosis, and predictors of mortality.

**Methods:**

This retrospective cohort study at a tertiary care hospital in Taiwan enrolled adult inpatients with first-episode splenic infarction diagnosed on computed tomography from January 2011 to May 2022. The primary outcome was 1-year all-cause mortality. Kaplan-Meier analysis and multivariate weighted Cox regression were applied for survival analysis.

**Results:**

The cohort included 304 individuals with an average age of 65.4 years. The mean CCI was 6, and active malignancy was observed in 49%. The estimated 1-year all-cause mortality rate was 61%, and the median survival time was 89 days. Compared with survivors, non-survivors exhibited significantly higher rates of left-sided pleural effusion, peri-splenic ascites, multiple or total infarction, and both portal and splenic vein thrombosis on CT imaging. Older age, active malignancy, decreased hemoglobin levels, low platelet counts, prolonged INR, elevated D-dimer, and peri-splenic ascites were independent predictors of mortality.

**Conclusion:**

In splenic infarction, 1-year mortality is notably high. The identified independent prognostic factors included age, malignancy, laboratory findings, and radiologic features. These findings may assist clinicians in early risk stratification for hospitalized patients with splenic infarction.

## Introduction

Splenic infarction is an uncommon disease that arises from diverse conditions, most commonly arrhythmia, infection, malignancy, acute aortic syndrome, and coagulation abnormalities 1-3. Patients typically exhibit a nonspecific presentation comprising left upper quadrant or left flank pain alongside leukocytosis, anemia, hypoalbuminemia, and elevations in lactate dehydrogenase and D-dimer 1, 4-6. Short-term mortality after splenic infarction has been relatively well characterized, with reported rates reaching 17-33% 1-3, 7. However, evidence on long-term prognosis remains limited.

Research regarding prognostic determinants of splenic infarction is also sparse. Our previous investigation focusing on short-term mortality in the emergency department (ED) population with splenic infarction identified uremia, malignancy, and high acuity as independent predictors 3. Nevertheless, the prognostic validity of these short-term predictors for long-term mortality has not been established. One cohort study has reported that a history of stroke or cirrhosis is associated with higher long-term all-cause mortality, whereas anticoagulation is associated with improved survival 8. However, these associations may lack robustness owing to the small sample size.

On contrast-enhanced computed tomography (CT), the hallmark feature is a wedge-shaped hypoattenuating parenchymal defect with its base abutting the splenic periphery. Other possible coexisting abnormalities include splenomegaly, ascites, portal vein thrombosis, splenic vein thrombosis, and infarction of other organs 9-11. However, the CT phenotype of these patients has not been systematically characterized, and its relevance to prognosis remains underexplored.

In these cases, the leading causes of mortality were attributable predominantly to malignancy, sepsis, respiratory tract infection, and infective endocarditis 3. While splenic infarction seldom directly accounts for death, numerous investigations have demonstrated its association with substantial subsequent mortality 1-3, 7. Despite this, its clinical importance and prognostic implications are often underestimated by healthcare providers and investigators. Given the paucity and limitations of the existing literature, this research aims to investigate the clinical and radiological characteristics and long-term prognosis of splenic infarction, and to identify predictors of one-year mortality. We seek to enhance recognition of the clinical significance and prognostic relevance of this disease among clinicians and researchers.

## Methods

### Study design and setting

This was a retrospective observational cohort study conducted at Taipei Veterans General Hospital, a tertiary care medical center in Taiwan. This hospital provides approximately 2,900 beds and reports an annual total of about 820,000 inpatient bed-days. All work in this investigation adhered to the principles of the Declaration of Helsinki. Institutional Review Board 2 of this hospital approved the study (approval number 2023-01-028BC), and informed consent was waived because of the retrospective and observational nature of the study design.

### Selection of participants

To screen the target population of interest, keywords related to splenic infarction were searched for in the CT report database. Inclusion criteria were hospitalized adult patients with first-episode splenic infarction diagnosed on CT during inpatient care or at the preadmission ED visit, with CT examination dates from January 2011 through May 2022. Exclusion criteria included the following: duplicate CT reports from different anatomical sites; CT performed outside the index hospitalization or the preadmission ED visit; age under 20 years; no evidence of splenic infarction; and traumatic splenic injury.

### Data collection

The medical records of eligible participants were accessed for the study from March 1, 2023, to October 1, 2023. Data on patient demographics and clinical characteristics were comprehensively reviewed in the electronic health record system. Age, sex, ED visit, comorbidities, pharmacologic therapy, symptoms, laboratory tests, co-infarction of other organs during the same hospitalization, surgeries, intensive care unit (ICU) admission, lengths of ICU and hospital stay, admission date, CT diagnosis date, and the last follow-up date were retrieved. A co-author and radiologist, YK, meticulously examined all CT images, confirmed the diagnosis of splenic infarction, and extracted imaging features 9-11.

Active malignancy was defined as any of the following: (1) a new or recently diagnosed cancer; (2) receipt of oncologic therapy in the prior six months; or (3) advanced-stage disease for which treatment options are constrained or infeasible 12, 13. The Charlson Comorbidity Index (CCI) was calculated based on their medical history 14.

### Outcomes

The primary outcome was one-year all-cause mortality. For analytic purposes, subjects were grouped as survivors versus non-survivors based on the last follow-up within one year.

### Statistical analysis

Continuous variables were reported as mean ± standard deviation (SD) and compared using Student's *t*-test. Categorical variables were presented as frequencies and percentages, with comparisons assessed by chi-square or Fisher's exact test. If missing values were present in laboratory variables, the variables were log-transformed and subjected to multiple imputation using the multivariate imputation by chained equations (MICE) framework with predictive mean matching. A total of m = 30 imputations were generated based on the proportion of missing data, with 20 iterations to ensure convergence and a fixed random seed to ensure reproducibility 15, 16. Relevant covariates, including clinical characteristics, laboratory tests, imaging findings, treatments, and prognosis from observed cases, were incorporated to predict missing values using a regression model-based approach. The imputed values were then back-transformed, and the resulting datasets were utilized for subsequent statistical modeling and analyses.

For survival analysis, Kaplan-Meier analysis was used to estimate the one-year all-cause mortality rate and median survival time. In addition, Schoenfeld residuals were used to evaluate the proportional hazards assumption. Weighted Cox regression using the Average Hazard Ratio (AHR) weighting approach, which assigns weights based on individuals' time at risk across event times to yield a time-averaged hazard ratio accommodating non-proportional hazards, was employed to identify prognostic factors for the primary outcome 17, 18. The time origin for survival analysis was defined as the date of CT diagnosis. A sensitivity analysis was additionally conducted using the date of hospital admission as the time origin, with adjustment for the interval from admission to CT diagnosis, to assess the robustness of the findings. For patients who received CT diagnosis in the emergency department before hospital admission, the admission-to-CT interval was defined as 0 days. In the univariate analysis, variables with *p* values below 0.05 were incorporated into the multivariate analysis employing backward elimination. Results were expressed in terms of hazard ratios (HRs) with 95% confidence intervals (CIs). The AHR can be interpreted as the average relative hazard over the entire follow-up period rather than a constant hazard ratio at any single time point. Because the proportional hazards assumption was not fully satisfied, AHRs provide a summary measure of the association between covariates and mortality over the follow-up period.

With respect to missing data, differences in the proportions of missing data between survivors and non-survivors were assessed using the chi-square test. Mortality rates were evaluated after stratification by missingness. Variables with missing values were analyzed prior to imputation. Furthermore, another sensitivity analysis based on complete-case Cox regression models was performed.

To address potential confounding by indication in the association between anticoagulant therapy and 1-year mortality, inverse probability of treatment weighting (IPTW) based on propensity scores was performed after multiple imputation as a sensitivity analysis. Propensity scores were estimated using baseline covariates that could influence both treatment allocation and mortality risk.

The dataset was collected with Microsoft Office Excel 2019. All statistical analyses were conducted using R (version 4.5.1) in the Google Colab environment. Multiple imputation was performed with the *mice* package, and survival analysis with the *coxphw* package. Statistical significance was defined as a two-sided *p* value < 0.05 in all analyses. The study was performed and documented in compliance with STROBE guidelines.

## Results

**Figure 1:**
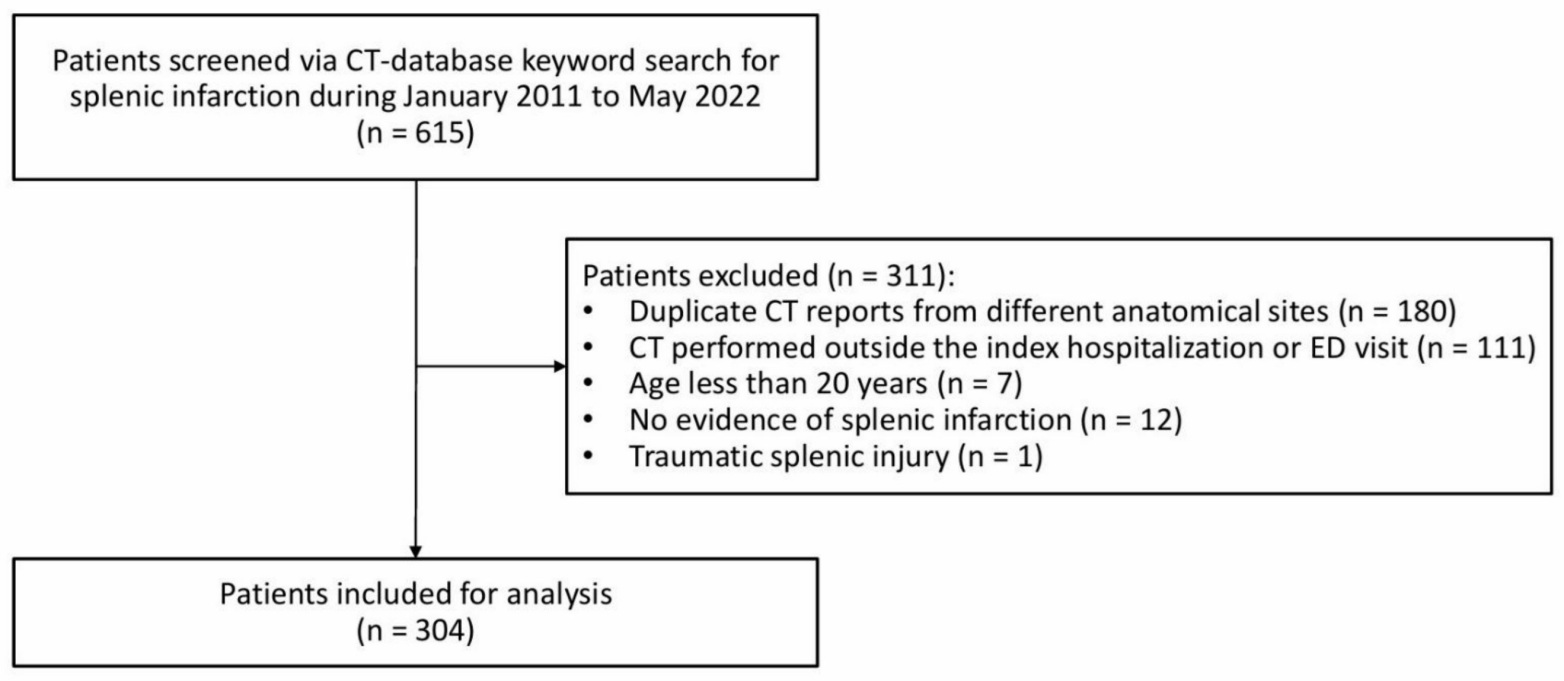
** Study participant enrollment flowchart**.

During the study period, 615 individuals were initially identified as eligible through keyword screening of the CT report database, and 304 patients were included in the final cohort after exclusions (Figure 1). As shown in Table 1, the study cohort had a mean age of 65.4 years, with 63.2% male. Admissions from the ED accounted for 21.4%. Common comorbidities included hypertension (51.3%), active malignancy (49.3%), and diabetes mellitus (35.5%), with a mean CCI of 6. In comparison to survivors, non-survivors showed significantly older age, greater prevalence of diabetes mellitus and active malignancy, and higher CCI. The pancreas, hematologic cancers, liver, and colorectum were the predominant sites of active malignancy among hospitalized patients with splenic infarction (Table 2).

The Kaplan-Meier analysis indicated an estimated 1-year all-cause mortality of 61% (95% CI: 55-66%), with a median survival time of 89 days (Figure S1). With respect to etiology and associated conditions, non-survivors exhibited higher proportions of active malignancy and sepsis, but lower proportions of acute aortic syndrome and unknown causes (Table 3).

Among cases, 7.2% experienced left upper quadrant abdominal pain and 34.2% reported abdominal pain in other locations. Fever (26.6%) and nausea/vomiting (14.1%) were also observed. Symptom distributions did not differ significantly between groups (Table 4).

Laboratory variables, including INR, APTT, D-dimer, albumin, BUN, total bilirubin, lactate dehydrogenase, and C-reactive protein, exhibited missing values. The proportion of missing data was highest for D-dimer, followed by lactate dehydrogenase and C-reactive protein. The proportion of missing albumin data was significantly higher in survivors than in non-survivors (p < 0.001), whereas no significant differences were observed for the other variables with missing values (Table S1 and Table S2). After multiple imputation, compared with survivors, non-survivors were characterized by lower levels of hemoglobin, platelet count, and albumin, and higher levels of INR, D-dimer, BUN, and total bilirubin (Table 4). In addition, an analysis of missing laboratory data prior to imputation is provided in Table S3, which showed similar findings and between-group differences compared with the results after multiple imputation.

**Figure 2:**
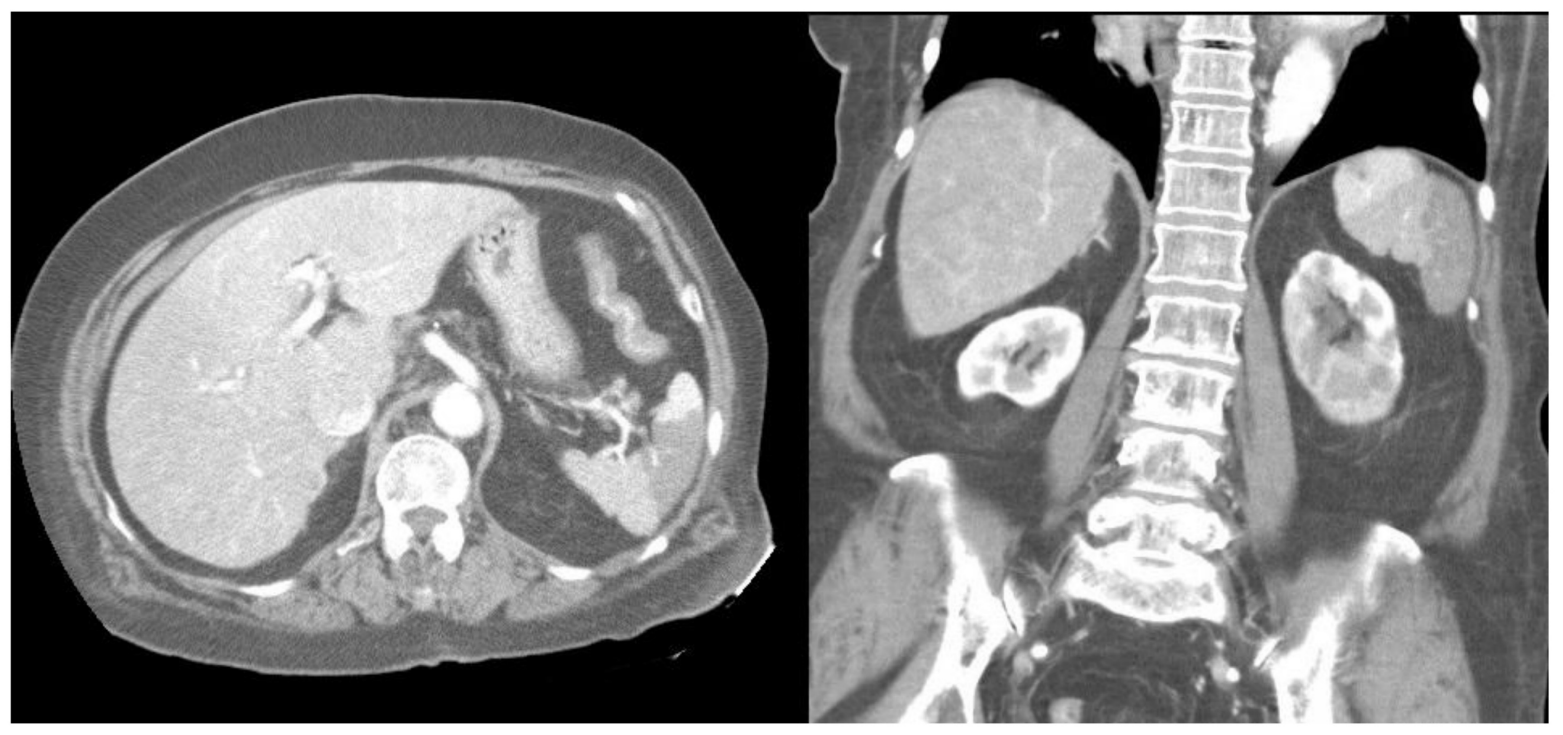
Concurrent splenic and left renal infarction. Contrast-enhanced abdominal CT images showing wedge-shaped hypodense regions within the spleen on the axial view (left panel) and within both the spleen and the left kidney on the coronal view (right panel).

**Figure 3:**
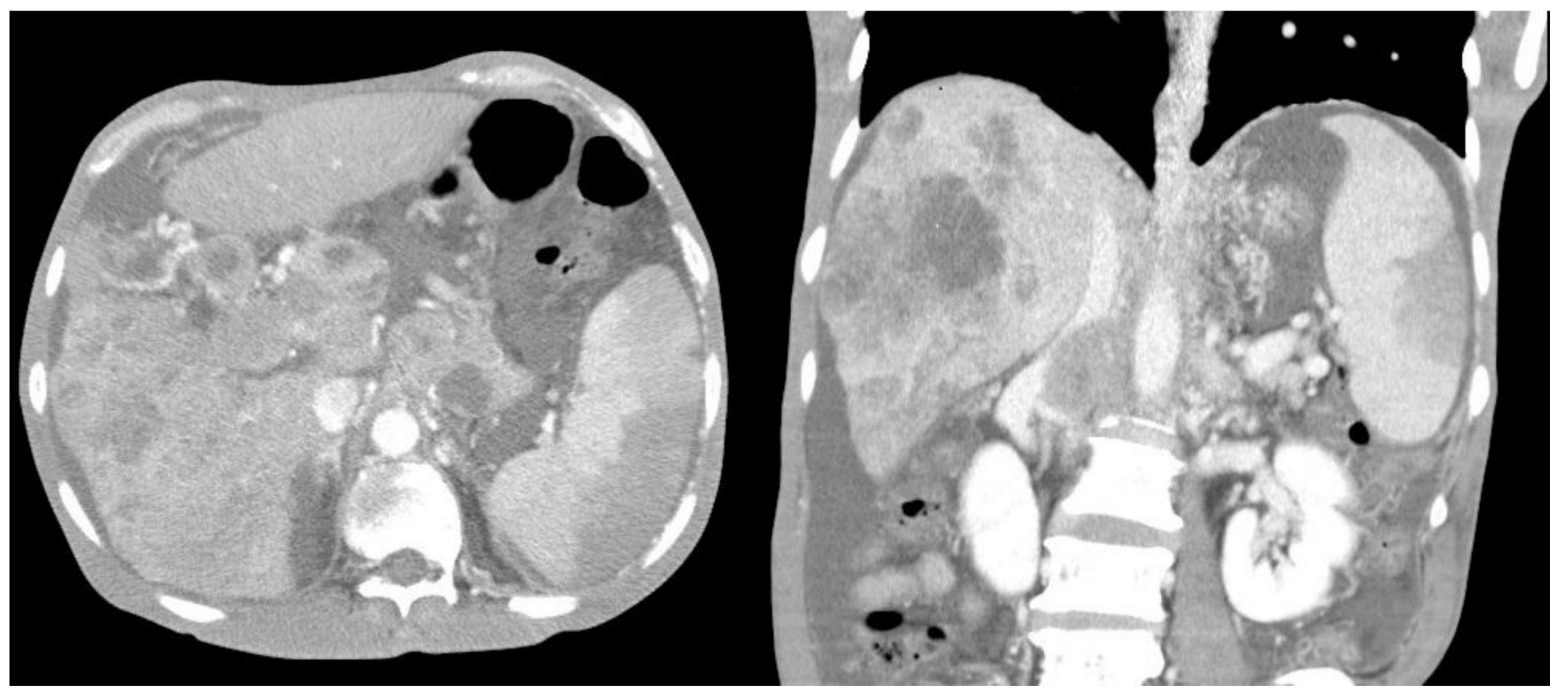
** Splenic infarction in a patient with hepatocellular carcinoma.** Contrast-enhanced abdominal CT reveals a wedge-shaped splenic hypodensity consistent with infarction, an ill-defined heterogeneous hypodense mass in the right hepatic lobe, portal vein thrombosis, and ascites.

Furthermore, compared with survivors, the non-survivors exhibited significantly higher rates of left-sided pleural effusion, peri-splenic ascites, multiple or total infarction, main portal vein thrombosis, and splenic vein thrombosis on CT imaging. Co-infarctions most commonly involved the kidneys, brain, and intestines. At least one organ co-infarction occurred in 30.6% of cases, while 11.5% experienced two or more, but no significant group differences were observed (Table S4). Figure 2 shows concurrent splenic and left renal infarction, whereas Figure 3 shows splenic infarction with portal vein thrombosis and ascites in a patient with hepatocellular carcinoma.

During hospitalization, the survivor group received more anticoagulants (Table 5). Overall, three patients (1%) underwent splenectomy. Study participants demonstrated prolonged ICU and hospital stays averaging 13 and 50 days, respectively. The leading causes of death included solid tumor malignancy (46%), hematologic malignancy (11%), sepsis or infectious disease (15%), intestinal or colonic ischemia (6%), and acute myocardial infarction or heart failure (5%) (Table S5).

The global test for the proportional hazards assumption, assessed using Schoenfeld residuals, was significant (p = 0.001), indicating that the assumption may not hold for the overall model. Several variables, including active malignancy, total bilirubin, splenic vein thrombosis, peri-splenic ascites, and anticoagulant use, showed evidence of violation of the proportional hazards assumption (Table S6). Weighted Cox regression using the AHR weighting approach was therefore performed to address the violation of the proportional hazards assumption. Univariate weighted Cox regression revealed several prognostic factors for 1-year mortality in splenic infarction. Multivariate weighted Cox regression analysis identified independent risk factors, including older age, active malignancy, decreased hemoglobin levels, low platelet counts, prolonged INR, elevated D-dimer, and peri-splenic ascites (Table 6).

In the sensitivity analysis using hospital admission as the time origin and adjusting for diagnostic delay, the main findings remained largely consistent. Active malignancy, age, platelet count, INR, D-dimer, and peri-splenic ascites remained significant predictors of 1-year mortality. In addition, BUN, multiple splenic infarctions, and total splenic infarction emerged as significant factors, whereas hemoglobin became insignificant (Table S7). In another sensitivity analysis using complete-case Cox regression models, active malignancy, hemoglobin, BUN, platelet count, and peri-splenic ascites were significantly associated with 1-year mortality (Table S8).

The unadjusted complete-case Cox regression and unadjusted weighted Cox regression using hospital admission as the time origin both identified anticoagulant use as a significant factor (HR 0.685, 95% CI 0.471-0.996, and HR 0.657, 95% CI 0.448-0.963, respectively). However, anticoagulant use was no longer statistically significant (HR 0.747, 95% CI 0.504-1.108) in the unadjusted weighted Cox regression when the CT diagnosis date was used as the time origin. Furthermore, IPTW analysis based on propensity scores after multiple imputation also showed no significant association (HR 1.143, 95% CI 0.758-1.720) (Table S9).

## Discussion

This retrospective cohort study demonstrated the clinical manifestations of splenic infarction in hospitalized patients, with a 1-year all-cause mortality rate of 61%. Multivariate weighted Cox regression analysis identified several independent mortality predictors, including older age, active malignancy, decreased hemoglobin levels, low platelet counts, prolonged INR, elevated D-dimer, and peri-splenic ascites.

A remarkably high 1-year mortality rate (61%) was observed in this study, together with prolonged ICU and hospital stays. These findings are likely attributable to multiple comorbidities (mean CCI 6) and a high prevalence of active malignancy (49.3%) in this cohort. Consistent with our findings, markedly high short-term mortality has been documented in several published articles 1-3, 7. The long-term mortality rate was described as relatively low, at 6.86 per 100 patient-years, in a multicenter study in Taiwan; however, that cohort excluded cases with active malignancy and hematologic illness and enrolled relatively younger participants than ours (61.0 vs. 65.4 years) 8. In addition, the leading causes of death, as shown in Table S5, were malignancy, severe infection, bowel ischemia, and acute heart disease among patients with splenic infarction. In this context, splenic infarction may act as a sentinel sign of catastrophic systemic microcirculatory failure or advanced abdominal conditions, rather than the direct cause of death.

Regarding the location of active malignancy, Table 2 shows that hepatobiliary, ampullary, and pancreatic malignancies accounted for about 40% of cases. This distribution may be related to local vascular compression caused by advanced tumors or regional lymphadenopathy. Besides, the pathophysiology of malignancy-related hypercoagulability (Trousseau syndrome) involves tumor cell-induced tissue factor production, mucin-producing activity, P- and L-selectin-mediated platelet-rich micro-thrombosis, tissue hypoxia, and oncogene activation, ultimately resulting in thrombin generation and fibrin deposition 19, 20. Both arterial and venous thromboembolism are prevalent in patients with cancer, a condition referred to as cancer-associated thrombosis (CAT), especially for pancreatic cancer, gastric cancer, lung cancer, and lymphoma. This thromboembolic diathesis significantly leads to morbidity and mortality 19, 21.

The classification of etiologies and associated conditions in Table 3 was based on published landmark literature and incorporated comorbidities, active illnesses, and imaging findings 1, 2, 4, 22. Apart from active malignancy, other etiologies and associated conditions, including cardioembolic diseases, sepsis, nonmalignant abdominal conditions, acute aortic syndromes or aneurysms, and connective tissue diseases, also appear to play important roles in patients with splenic infarction. In addition, because patients with acute aortic syndrome who survived to hospitalization may be subject to confounding by indication, this variable was not included in the regression analysis.

Our weighted Cox regression model demonstrated active malignancy as an independent predictor of mortality. Active malignancy was most commonly located in the pancreas, hematologic system, liver, and colorectum. Splenic infarction is possibly attributable to hypercoagulation or local invasion into the hepatobiliary system or splenic vasculature among cancer patients 10, 23, 24. Consistently, malignancy has also been described as a prognostic factor for short-term mortality, and this research extended the evidence by validating its relevance to long-term prognosis 3, 22. Because malignancy is included in the CCI, a modified CCI was calculated by excluding malignancy-related components to avoid conceptual overlap and potential collinearity with the active malignancy variable in the regression models. After adjustment for the modified CCI, active malignancy remained an independent prognostic factor. Accordingly, our findings indicate that cancer patients presenting with splenic infarction should be considered for earlier prognostic evaluation, with timely management of the underlying malignancy may help improve clinical outcomes.

Given that the proportional hazards assumption was not satisfied for several variables in this study, a weighted Cox regression model using the AHR weighting approach was employed. This approach yields a time-averaged hazard ratio, enabling valid inference under non-proportional hazards. AHRs were used to summarize the association between covariates and mortality over the entire 1-year follow-up period. For instance, active malignancy was associated with an AHR of 1.873, indicating that patients with active malignancy had an approximately 1.9-fold higher average hazard of death during the 1-year follow-up period. Consequently, the reported hazard ratios represent weighted averages over the full follow-up period, rather than assuming a constant effect over time. While this approach improves model validity, the temporal patterns of risk associated with certain variables may not be fully captured. Future well-designed studies incorporating time-dependent covariates or flexible survival models are warranted to better elucidate these dynamic relationships.

The missingness of albumin differed significantly between survivors and non-survivors, suggesting that its missing mechanism may not be completely random. This may reflect clinical practice in which albumin is more frequently measured in critically ill patients. This suggests that albumin has clinical importance. Although the assumption of missing at random (MAR) may not fully hold for this variable, the missingness of most other variables can reasonably be considered consistent with the MAR assumption. Given that albumin is likely associated with disease severity, its missingness may introduce bias. Nevertheless, the use of multiple imputation to handle missing values and the inclusion of these variables as potential prognostic factors in multivariable regression for adjustment are considered appropriate. Complete-case analysis may reduce statistical power and introduce selection bias due to missing data, whereas multiple imputation makes more efficient use of available data. The analysis of laboratory data following multiple imputation yielded results consistent with those observed prior to imputation, particularly regarding between-group differences and statistical significance (Table S3), thereby supporting the validity of the multiple imputation approach. In the sensitivity analysis using a complete-case Cox regression model (Table S8), the overall findings were generally consistent with the main results from the multivariate weighted Cox regression model (Table 6), supporting the robustness of our findings.

Low platelet counts, prolonged INR, and elevated D-dimer were identified as independent prognostic factors in this investigation. Taken together, these three factors suggest coagulopathy, with a tendency toward liver failure or disseminated intravascular coagulation (DIC) 25. With elevated serum total bilirubin (mean 2.9 mg/dL) and a high prevalence of ascites (41.8%) on CT imaging in our cohort, liver decompensation might be considered one of the predictors. Nevertheless, serum total bilirubin was not an independent risk factor in our final model, and the role of liver failure remains to be clarified. DIC, frequently induced by critical illnesses such as trauma, malignancy, sepsis, and obstetric complications, results in the paradoxical manifestation of both thrombosis and bleeding owing to coagulation factor consumption and leads to a critical clinical condition and poor prognosis 26, 27.

Anticoagulant use has been reported to be associated with reduced long-term mortality in patients with splenic infarction in prior research 8, which is consistent with our findings from unadjusted complete-case Cox regression and unadjusted weighted Cox regression using hospital admission as the time origin. However, this apparent association in these models may be subject to bias, including missing data, selection bias, immortal time bias, and confounding by indication. To reduce potential bias related to missing data, selection bias, and immortal time bias, we used the CT diagnosis date as the time origin after multiple imputation. In this analysis, unadjusted weighted Cox regression showed no significant association between anticoagulant use and 1-year mortality. Patients receiving anticoagulants may have had more favorable baseline characteristics, such as lower disease severity or reduced bleeding risk, which could contribute to better outcomes; thus, confounding by indication remains a potential concern. Therefore, IPTW based on propensity scores was applied as a sensitivity analysis to better estimate the effect of anticoagulation on survival, and no statistically significant association was observed. This analysis supports the robustness of our main finding that anticoagulant use was not an independent prognostic factor.

In the sensitivity analysis using the date of hospital admission as the time origin and adjusting for the interval from hospital admission to CT diagnosis (Table S7), the main findings remained largely consistent, supporting the robustness of the identified prognostic factors in the primary analysis. Although non-survivors exhibited a longer admission-to-CT interval in descriptive analyses (Table 4), this association was reversed in time-to-event analyses, including univariate weighted Cox regression. This discrepancy likely arises from differences in analytical approaches, as descriptive analyses ignore event timing and censoring, whereas Cox regression accounts for time-to-event dynamics and risk sets. The admission-to-CT interval varied across patients and likely reflected differences in clinical severity. Patients with more severe presentations may undergo earlier imaging, whereas those with less specific or milder symptoms may experience delayed diagnosis. Nevertheless, the association between hemoglobin level and mortality was attenuated after accounting for diagnostic delay, suggesting potential confounding by diagnostic timing and disease severity. Patients with severe anemia may undergo earlier imaging, potentially introducing bias if not accounted for. The identification of multiple or total splenic infarction as significant predictors in the sensitivity analysis may also reflect improved adjustment for disease severity through inclusion of the admission-to-CT interval. These findings highlight the importance of accounting for diagnostic timing in observational studies of acute conditions and may help explain the observed differences in certain variables between the primary and sensitivity analyses, particularly given the influence of time-to-event dynamics and potential confounding.

Our results revealed no significant association between fatal outcomes and serum creatinine, while a significant association was observed with serum BUN in the sensitivity analysis. Previous evidence has suggested that a higher BUN-to-creatinine ratio may be linked to longer ICU stays and increased in-hospital mortality 28, 29. Serum BUN elevation could primarily represent muscle protein catabolism in the context of prolonged critical illness, but it may also arise from dehydration, gastrointestinal hemorrhage, severe infection, or impaired renal and hepatic function 29, 30. Cardiorenal syndrome may also contribute to increased circulating BUN levels. In severe heart disease or cardiac decompensation, reduced cardiac output and impaired renal perfusion may activate the renin-angiotensin-aldosterone system and stimulate the sympathetic nervous system. These neurohormonal responses enhance renal tubular urea reabsorption, which may subsequently result in elevated serum BUN levels 31. The comorbidities and associated diseases of splenic infarction—such as cancer, infective endocarditis, sepsis, and severe heart failure—may help elucidate the underlying causes of BUN elevation 3. Furthermore, this finding emphasizes that splenic infarction in the context of severe underlying disease should prompt increased clinical awareness and monitoring.

Our study comprehensively reviewed the radiological features of splenic infarction on CT. Our observations were consistent with prior research, demonstrating comparable prevalences of peri-splenic ascites, left-sided pleural effusion, and multiple infarcts 6. In univariate weighted Cox regression analysis, left-sided pleural effusion, peri-splenic ascites, multiple or total splenic infarction, and main portal vein thrombosis were significant. Furthermore, our research demonstrated that the presence of peri-splenic ascites was significantly and independently correlated with mortality after adjustment. The presence of ascites typically indicates an underlying advanced systemic or local disorder, pointing to critical derangements in fluid homeostasis, portal hypertension, or peritoneal integrity, primarily including cirrhosis, malignant ascites, right-sided heart failure, and nephrotic syndrome 32. Consequently, the prognostic relevance of this radiological finding highlights the critical role of peri-splenic ascites in splenic infarction and emphasizes it as a key feature warranting attention in future imaging evaluations.

In this study, concomitant infarction of other organs was observed in 30.6% of patients with splenic infarction during the same hospitalization, particularly renal infarction, mesenteric ischemia, and cerebral infarction. Additionally, 11.5% of patients had infarctions in two or more other organs. The high prevalence of co-infarction observed in our study is consistent with prior literature 4, 9. These concurrent thrombotic events may indicate systemic embolization or a thrombotic storm and may be attributable to severe sepsis, DIC, advanced malignancy, or aortic dissection 2, 13, 33.

Although conducted at a single medical center in Taiwan, this research enrolled 304 individuals over approximately 12 years. To our knowledge, it represents the largest comprehensively analyzed cohort to date. Bewersdorf et al focused on 281 cancer patients out of 591 splenic infarction cases screened in the USA 23. Of 353 splenic infarction patients identified in Korea, Im and colleagues concentrated on 101 infected subjects 33. Furthermore, our investigation thoroughly reviewed clinical and radiological characteristics, addressed long-term prognosis, and examined predictors of mortality after multiple imputation and multivariate adjustment. For clinical application, early risk identification may be considered for patients characterized by advanced age, active malignancy, coagulopathy, anemia, or ascites. From a research perspective, additional prospective multicenter investigations are required to explore reversible prognostic factors for splenic infarction and to improve patient outcomes.

This research had several limitations. First, this study was retrospective in design and involved missing laboratory data. The missingness of certain laboratory variables, particularly albumin, may not fully satisfy the assumption of missing at random, thereby introducing potential bias even after multiple imputation. Second, eligible participants were derived exclusively from a single hospital. Third, this study focused on hospitalized patients with CT-confirmed splenic infarction, excluding outpatients as well as mild or asymptomatic cases that may have been managed conservatively in the ED settings without hospital admission or imaging confirmation. Consequently, our study population may represent patients with relatively more severe clinical presentations, which may limit the generalizability of our findings to the broader population with splenic infarction. Given the above limitations, well-designed multicenter prospective studies with comprehensive data collection are warranted to minimize bias, identify key prognostic factors, and enhance the generalizability.

## Conclusion

In summary, the 1-year mortality rate among hospitalized adult inpatients with splenic infarction was considerably high, reaching 61%. For clinical practice, early risk assessment and prognostic counseling should be considered for hospitalized patients with advanced age, active malignancy, coagulopathy, anemia, or peri-splenic ascites. For investigators, prospective multicenter studies with robust designs are warranted to enable comprehensive analysis, identify modifiable factors, and ultimately improve clinical outcomes.

## Supplementary Material

Supplementary figure and tables.

## Figures and Tables

**Table 1:** Baseline characteristics among hospitalized patients with splenic infarction

Characteristics	All (n = 304)	Survivors (n = 126)	Non-survivors (n = 178)	*p* value
Age	65.4 ± 16.1	62.4 ± 17.6	67.6 ± 14.8	0.007
Male sex	192 (63.2)	82 (65.1)	110 (61.8)	0.559
Admission from ED	65 (21.4)	32 (25.4)	33 (18.5)	0.151
Comorbidities				
Dyslipidemia	39 (12.8)	16 (12.7)	23 (12.9)	0.954
Hypertension	156 (51.3)	69 (54.8)	87 (48.9)	0.312
Diabetes mellitus	108 (35.5)	34 (27.0)	74 (41.6)	0.009
Coronary artery disease	60 (19.7)	24 (19.0)	36 (20.2)	0.799
Valvular heart disease	27 (8.9)	12 (9.5)	15 (8.4)	0.741
Myocardial infarction	8 (2.6)	4 (3.2)	4 (2.2)	0.722
Congestive heart failure	37 (12.2)	15 (11.9)	22 (12.4)	0.905
Active malignancy	150 (49.3)	38 (30.2)	112 (62.9)	< 0.001
CVA or TIA	38 (12.5)	12 (9.5)	26 (14.6)	0.187
Uremia requiring renal replacement therapy	27 (8.9)	8 (6.3)	19 (10.7)	0.192
Liver cirrhosis	35 (11.5)	14 (11.1)	21 (11.8)	0.853
COPD	19 (6.3)	9 (7.1)	10 (5.6)	0.588
PAOD	12 (3.9)	4 (3.2)	8 (4.5)	0.767
CCI	6 ± 4	5 ± 3	7 ± 3	< 0.001
Medications use before hospitalization				
Antiplatelet drugs	48 (15.8)	24 (19.0)	24 (13.5)	0.190
Anticoagulants	31 (10.2)	18 (14.3)	13 (7.3)	0.047

*ED: emergency department; CVA: c*erebrovascular accident; TIA: *transient ischemic attack;* COPD: c*hronic obstructive pulmonary disease;* PAOD: peripheral arterial occlusive disease; CCI: Charlson Comorbidity Index

**Table 2:** Location of active malignancy among hospitalized patients with splenic infarction

Location	All (n = 150)	Survivors (n = 38)	Non-survivors (n = 112)	*p* value
Head and neck^1^	5 (3%)	1 (3%)	4 (4%)	1.000
Esophagus	4 (3%)	3 (8%)	1 (1%)	0.050
Stomach	6 (4%)	2 (5%)	4 (4%)	0.643
Small intestine	2 (1%)	1 (3%)	1 (1%)	0.444
Colon or rectum	13 (9%)	6 (16%)	7 (6%)	0.094
Liver^3^	17 (11%)	3 (8%)	14 (13%)	0.562
Biliary tract cancer	10 (7%)	2 (5%)	8 (7%)	1.000
Ampulla of Vater	3 (2%)	1 (3%)	2 (2%)	1.000
Pancreas	30 (20%)	5 (13%)	25 (22%)	0.222
Urinary tract	6 (4%)	2 (5%)	4 (4%)	0.643
Prostate	4 (3%)	1 (3%)	3 (3%)	1.000
Uterus or ovary	8 (5%)	5 (13%)	3 (3%)	0.025
Leukemia^2^	9 (6%)	0 (0%)	9 (8%)	0.112
Lymphoma^1^	18 (12%)	6 (16%)	12 (11%)	0.398
Multiple myeloma	2 (1%)	0 (0%)	2 (2%)	1.000
Lung^3^	11 (7%)	0 (0%)	11 (10%)	0.066
Others (skin, soft tissue, vessel)^2^	4 (3%)	0 (0%)	4 (4%)	0.572
Unknown primary origin	1 (1%)	0 (0%)	1 (1%)	1.000

^1^One patient had concurrent tonsillar cancer and lymphoma. ^2^Another patient had concurrent fibromyxosarcoma of the limb and acute myeloid leukemia. ^3^Another patient had concurrent lung cancer and hepatocellular carcinoma.

**Table 3:** Etiology and associated conditions of splenic infarction

Etiology and associated conditions	All (n = 304)	Survivors (n = 126)	Non-survivors (n = 178)	*p* value
Active malignancy^1^	150 (49.3)	38 (30.2)	112 (62.9)	<0.001
Abdominal solid tumor	99 (32.6)	28 (22.2)	71 (39.9)	0.001
Non-abdominal solid tumor	25 (8.2)	4 (3.2)	21 (11.8)	0.007
Hematologic malignancy	29 (9.5)	6 (4.8)	23 (12.9)	0.017
Cardioembolic diseases	98 (32.2)	39 (31.0)	59 (33.1)	0.687
Atrial fibrillation	34 (11.2)	15 (11.9)	19 (10.7)	0.737
Infective endocarditis	17 (5.6)	10 (7.9)	7 (3.9)	0.134
Others^2^	82 (27.0)	32 (25.4)	50 (28.1)	0.602
Sepsis^3^	68 (22.4)	19 (15.1)	49 (27.5)	0.010
Nonmalignant abdominal conditions^4^	118 (38.8)	41 (32.5)	77 (43.1)	0.059
Hypercoagulable state^5^	17 (5.6)	10 (7.9)	7 (3.9)	0.134
Atherosclerosis of abdominal aorta, celiac trunk, or splenic artery	24 (7.9)	14 (11.1)	10 (5.6)	0.080
Aortic dissection or aneurysm	24 (7.9)	19 (15.1)	5 (2.8)	<0.001
Iatrogenic^6^	22 (7.2)	6 (4.8)	16 (9.0)	0.161
Autoimmune disease	11 (3.6)	4 (3.2)	7 (3.9)	1.000
Unknown cause	11 (3.6)	10 (7.9)	1 (0.6)	<0.001

^1^ Three patients had concurrent malignancies: (1) tonsillar cancer and lymphoma; (2) fibromyxosarcoma of the limb and acute myeloid leukemia; and (3) lung cancer and hepatocellular carcinoma. ^2^ Other cardioembolic diseases included arrhythmias, severe heart failure, myocardial infarction, nonbacterial thrombotic endocarditis, and pulmonary arterial hypertension. ^3^ Sepsis in this table excluded infections limited to infective endocarditis and intra-abdominal sources. ^4^ Nonmalignant abdominal conditions included pancreatitis, liver cirrhosis, bowel ischemia, recent abdominal surgery, severe gastrointestinal bleeding, severe intra-abdominal bleeding, peritonitis, severe colitis, cholangitis, and cholecystitis. ^5^ Hypercoagulable states included antithrombin III deficiency, myeloproliferative neoplasms, essential thrombocytosis, thrombotic thrombocytopenic purpura, and simultaneous vascular thrombosis (e.g., splenic vein thrombosis, portal vein thrombosis, deep vein thrombosis, and pulmonary embolism) in the absence of other identifiable causes. ^6^ Iatrogenic causes included recent aortic intervention, extracorporeal membrane oxygenation (ECMO), or embolization of the celiac trunk or its branches.

**Table 4:** Clinical manifestations among hospitalized patients with splenic infarction

Variables	All (n = 304)	Survivors (n = 126)	Non-survivors (n = 178)	*p* value
Symptoms				
Abdominal pain in LUQ location	22 (7.2)	13 (10.3)	9 (5.1)	0.081
Abdominal pain in other location	104 (34.2)	46 (36.5)	58 (32.6)	0.477
Left flank pain	10 (3.3)	3 (2.4)	7 (3.9)	0.531
Fever	81 (26.6)	32 (25.4)	49 (27.5)	0.679
Nausea or vomiting	43 (14.1)	17 (13.5)	26 (14.6)	0.784
Laboratory examination				
*White blood cell count* (/μL)	12562 ±11341	12193 ± 11038	12823 ± 11574	0.631
Hemoglobin (g/dL)	10.0 ± 2.5	10.8 ± 2.5	9.4 ± 2.2	<0.001
Platelet (x10^3^/μL)	180 ± 155	233 ± 182	142 ± 120	<0.001
INR	1.4 ± 0.8	1.2 ± 0.4	1.5 ± 1.0	<0.001
APTT (seconds)	35 ± 20	33 ± 16	37 ± 22	0.099
D-dimer (μg/mL)	13.1 ± 19.8	9.4 ± 14.9	15.8 ± 22.2	0.014
Albumin (g/dL)	3.0 ± 0.6	3.2 ± 0.6	2.9 ± 0.6	<0.001
BUN (mg/dL)	30 ± 27	26 ± 21	33 ± 30	0.011
Creatinine (mg/dL)	1.5 ± 1.6	1.6 ± 1.8	1.5 ± 1.4	0.421
Total bilirubin (mg/dL)	2.9 ± 5.1	1.9 ± 3.9	3.6 ± 5.7	0.003
Lactate dehydrogenase (IU/L)	590 ± 1083	544 ± 1025	623 ± 1118	0.559
C-reactive protein (mg/dL)	12.1 ± 42.6	8.1 ± 9.9	14.9 ± 54.5	0.124
Findings in computed tomography				
Left-sided pleural effusion	189 (62.2)	69 (54.8)	120 (67.4)	0.025
Peri-splenic ascites	146 (48.0)	50 (39.7)	96 (53.9)	0.014
Infarction pattern				0.027
Single splenic infarction	96 (31.6)	50 (39.7)	46 (25.8)	
Multiple splenic infarction	193 (63.5)	72 (57.1)	121 (68.0)	
Total splenic infarction	15 (4.9)	4 (3.2)	11 (6.2)	
Infarction size (largest diameter, mm)	43 ± 27	44 ± 31	43 ± 25	0.776
Spleen size				
Axial (mm)	111 ± 29	113 ± 30	110 ± 28	0.335
Coronal oblique (mm)	115 ± 34	119 ± 41	112 ± 28	0.121
Cirrhotic liver	73 (24.0)	24 (19.0)	49 (27.5)	0.088
Main portal vein thrombosis	22 (7.2)	4 (3.2)	18 (10.1)	0.021
Splenic vein thrombosis	32 (10.5)	5 (4.0)	27 (15.2)	0.002
Spleen rupture	2 (0.7)	1 (0.8)	1 (0.6)	1.000
Admission-to-CT Interval (days)	11.8 ± 21.2	7.9 ± 11.8	14.5 ± 25.5	0.002

LUQ: left upper quadrant; INR: international normalized ratio*; APTT:* activated partial thromboplastin time; BUN: Blood urea nitrogen; CT: computed tomography

**Table 5:** Therapy and prognosis among hospitalized patients with splenic infarction

Variables	All (n = 304)	Survivors (n = 126)	Non-survivors (n = 178)	*p* value
Pharmacologic therapy^*^				
Anticoagulants	71 (23.4)	37 (29.4)	34 (19.1)	0.037
Antiplatelet agents	87 (28.6)	41 (32.5)	46 (25.8)	0.203
Analgesics	179 (58.9)	66 (52.4)	113 (63.5)	0.053
Splenectomy	3 (1.0)	3 (2.4)	0 (0.0)	0.070
Length of stay				
ICU (days)	13 ± 32	10 ± 20	16 ± 39	0.067
Hospitalization (days)	50 ± 216	65 ± 329	39 ± 55	0.389

^*^Medications encompassed both the patients' existing treatments and newly initiated pharmacological therapies. ICU: intensive care unit

**Table 6:** Weighted Cox regression analysis of factors associated with 1-year mortality among hospitalized patients with splenic infarction

Predictive Variables	Univariate analysis		Multivariate analysis^*^	
	HR^**^ (95% CI)	*p* value	HR^**^ (95% CI)	*p* value
Age	1.011 (1.002-1.020)	0.021	1.019 (1.008-1.030)	< 0.001
Diabetes mellitus	1.327 (0.985-1.788)	0.063		
Active malignancy	1.855 (1.354-2.542)	< 0.001	1.873 (1.360-2.580)	< 0.001
Modified CCI^***^	1.055 (1.003-1.109)	0.038		
Sepsis	1.402 (1.019-1.930)	0.038		
Unknown cause	0.170 (0.022-1.310)	0.089		
Hemoglobin	0.869 (0.816-0.927)	< 0.001	0.911 (0.852-0.974)	0.006
Platelet	0.997 (0.995-0.998)	< 0.001	0.997 (0.996-0.999)	< 0.001
INR	1.214 (1.075-1.371)	0.002	1.198 (1.063-1.349)	0.003
D-dimer	1.011 (1.003-1.019)	0.007	1.010 (1.002-1.017)	0.010
Albumin	0.651 (0.514-0.823)	< 0.001		
BUN	1.007 (1.003-1.011)	0.001		
Total bilirubin	1.052 (1.018-1.088)	0.003		
Left-sided pleural effusion	1.546 (1.125-2.123)	0.007		
Peri-splenic ascites	1.740 (1.294-2.341)	< 0.001	1.714 (1.275-2.305)	< 0.001
Multiple splenic infarction	1.507 (1.068-2.127)	0.020		
Total splenic infarction	2.856 (1.337-6.136)	0.007		
Main portal vein thrombosis	1.814 (1.137-2.894)	0.012		
Splenic vein thrombosis	1.294 (0.931-1.800)	0.125		
Anticoagulant therapy	0.747 (0.504-1.108)	0.147		

^*^ Variables with p < 0.05 in univariate analyses were entered into multivariate analysis using backward elimination. ^**^HR denotes the average hazard ratio (AHR) estimated using weighted Cox regression that accommodates potential non-proportional hazards. ^***^Because malignancy is included in the CCI, a modified CCI was calculated by excluding malignancy-related components to avoid conceptual overlap and potential collinearity with the active malignancy variable in the model. CI: confidence interval; INR: international normalized ratio*;* BUN: blood urea nitrogen

## Data Availability

The authors confirm that the data supporting the findings of this study are available within the article and its supplementary materials.

## Abbreviations

ED: emergency department
CT: computed tomography
ICU: intensive care unit
CCI: Charlson comorbidity index
SD: standard deviation
MICE: multivariate imputation by chained equations
AHR: average hazard ratio
HR: hazard ratio
CI: confidence interval
STROBE: strengthening the reporting of observational studies in epidemiology
INR: international normalized ratio
BUN: blood urea nitrogen
DIC: disseminated intravascular coagulation
