# Supplementary material for: Predictors of One-Year Mortality in Hospitalized Patients with Splenic Infarction: Survival Analysis of a Retrospective Cohort in Taiwan
Source: Int J Med Sci. 2026 Apr 23;23(6):2016–26. doi: 10.7150/ijms.130149 (PMC13181384; doi:10.7150/ijms.130149)
Supplement: Supplementary file 1 — Supplementary figure and tables. [file ijmsv23p2016s1.pdf]

Table S1. Proportions of Missing Laboratory Measurements among Hospitalized Patients with Splenic Infarction

Variables	All (n = 304)	Survivors (n = 126)	Non-survivors (n = 178)	*p* value
INR	14 (4.6)	7 (5.6)	7 (3.9)	0.698
APTT	14 (4.6)	7 (5.6)	7 (3.9)	0.698
D-dimer	93 (30.6)	45 (35.7)	48 (27.0)	0.133
Albumin	23 (7.6)	16 (12.7)	7 (3.9)	<0.001
BUN	14 (4.6)	7 (5.6)	7 (3.9)	0.698
Total bilirubin	18 (5.9)	10 (7.9)	8 (4.5)	0.314
Lactate dehydrogenase	65 (21.4)	29 (23.0)	36 (20.2)	0.658
C-reactive protein	33 (10.9)	17 (13.5)	16 (9.0)	0.291

INR: international normalized ratio; APTT: activated partial thromboplastin time; BUN: Blood urea nitrogen

Table S2. Comparison of Mortality Rates between Patients with Missing and Non-Missing Data

Variables	Patients with missing data		Patients without missing data		*p* value
	Number (%)	Mortality rate	Number (%)	Mortality rate	
INR	14 (4.6)	50.0%	290 (95.4)	59.0%	0.698
APTT	14 (4.6)	50.0%	290 (95.4)	59.0%	0.698
D-dimer	93 (30.6)	51.6%	211 (69.4)	61.6%	0.133
Albumin	23 (7.6)	30.4%	281 (92.4)	60.9%	<0.001
BUN	14 (4.6)	50.0%	290 (95.4)	59.0%	0.698
Total bilirubin	18 (5.9)	44.4%	286 (94.1)	59.4%	0.314
Lactate dehydrogenase	65 (21.4)	55.4%	239 (78.6)	59.4%	0.658
C-reactive protein	33 (10.9)	48.5%	271 (89.1)	59.8%	0.291

INR: international normalized ratio; APTT: activated partial thromboplastin time; BUN: Blood urea nitrogen

Table S3. Analysis of Missing Laboratory Data Prior to Imputation among Hospitalized Patients with Splenic Infarction

Variables (number of available observations)	All	Survivors	Non-survivors	*p* value
INR (n=290)	1.4 ± 0.8	1.2 ± 0.4	1.5 ± 1.0	<0.001
APTT (seconds) (n=290)	35 ± 20	33 ± 16	37 ± 22	0.105
D-dimer (µg/mL) (n=211)	13.3 ± 19.8	9.3 ± 14.2	15.9 ± 22.2	0.009
Albumin (g/dL) (n=281)	3.0 ± 0.6	3.2 ± 0.6	2.9 ± 0.6	0.001
BUN (mg/dL) (n=290)	30.4 ± 26.9	25.9 ± 20.7	33.6 ± 30.1	0.011
Total bilirubin (mg/dL) (n=286)	2.9 ± 5.2	2.0 ± 4.0	3.6 ± 5.8	0.005
Lactate dehydrogenase (IU/L) (n=239)	603 ± 1111	560 ± 1090	633 ± 1128	0.619
C-reactive protein (mg/dL) (n=271)	12.7 ± 44.5	8.3 ± 8.4	12.8 ± 57	0.102

INR: international normalized ratio; APTT: activated partial thromboplastin time; BUN: Blood urea nitrogen

Table S4. Distribution of Co-Infarctions in Other Organs among Hospitalized Patients with Splenic Infarction

Variables	All (n = 304)	Survivors (n = 126)	Non-survivors (n = 178)	*p* value
Kidney	39 (12.8)	21 (16.7)	18 (10.1)	0.092
Brain	36 (11.8)	10 (7.9)	26 (14.6)	0.076
Intestine	28 (9.2)	9 (7.1)	19 (10.7)	0.294
Liver	13 (4.3)	3 (2.4)	10 (5.6)	0.169
Lung	13 (4.3)	2 (1.6)	11 (6.2)	0.051
Heart	6 (2.0)	2 (1.6)	4 (2.2)	1.000
Pancreas	4 (1.3)	1 (0.8)	3 (1.7)	0.644
Limb	4 (1.3)	1 (0.8)	3 (1.7)	0.644
Adrenal gland	1 (0.3)	0 (0.0)	1 (0.6)	1.000
Stomach	1 (0.3)	0 (0.0)	1 (0.6)	1.000
Co-infarction of one or more organs	93 (30.6)	38 (30.2)	55 (30.9)	0.890
Co-infarction of two or more organs	35 (11.5)	10 (7.9)	25 (14.0)	0.100

Table S5. Causes of Death among Hospitalized Patients with Splenic Infarction

Causes of mortality	Non-survivors (n = 178)
Solid tumor malignancy	81 (46%)
Hematologic malignancy	20 (11%)
Sepsis or infectious disease[1]	27 (15%)
Acute myocardial infarction or heart failure	9 (5%)
Pulmonary arterial hypertension	2 (1%)
Other pulmonary condition[2]	2 (1%)
Acute aortic syndrome	3 (2%)
Cerebral infarction	3 (2%)
Intestinal or colonic ischemia	11 (6%)
Gastro-intestinal tract bleeding	3 (2%)
Liver cirrhosis or liver failure	5 (3%)
Other intra-abdominal disease[3]	6 (3%)
Myelodysplastic syndrome	2 (1%)
Trauma or burn injury	2 (1%)
Unknown	2 (1%)

[1] including pneumonia and infective endocarditis

[2] including acute respiratory distress syndrome and acute respiratory failure

[3] including peritonitis, Intraabdominal abscess, pancreatitis, and hepatic artery rupture

Table S6. Assessment of the Proportional Hazards Assumption Using Schoenfeld Residuals in Hospitalized Patients with Splenic Infarction

Variable	χ^2	df	*p* value
GLOBAL	43.700	19	0.001
Age	3.070	1	0.080
Diabetes mellitus	2.150	1	0.142
Active malignancy	10.400	1	0.001
Modified CCI*	0.006	1	0.936
Sepsis	1.380	1	0.240
Unknown cause	2.850	1	0.091
Hemoglobin	1.540	1	0.215
Platelet	0.172	1	0.678
INR	0.021	1	0.884
D-dimer	0.218	1	0.640
Albumin	0.942	1	0.332
BUN	1.330	1	0.249
Total bilirubin	5.800	1	0.016
Left-sided pleural effusion	0.315	1	0.575
Peri-splenic ascites	4.63	1	0.031
Multiple or total splenic infarction	0.360	1	0.549
Main portal vein thrombosis	0.000	1	0.991
Splenic vein thrombosis	12.000	1	<0.001
Anticoagulant therapy	7.730	1	0.005

*Because malignancy is included in the CCI, a modified CCI was calculated by excluding malignancy-related components to avoid conceptual overlap and potential collinearity with the active malignancy variable in the model.

INR: international normalized ratio; BUN: blood urea nitrogen

Table S7. Sensitivity Analysis of a Weighted Cox Regression Model Using Hospital Admission as the Time Origin, with Adjustment for the Admission-to-CT Interval, for Factors Associated with 1-Year Mortality in Hospitalized Patients with Splenic Infarction

Predictive Variables	Univariate analysis		Multivariate analysis*	
	HR** (95% CI)	*p* value	HR** (95% CI)	*p* value
Age	1.013 (1.00–1.022)	0.003	1.022 (1.012–1.033)	<0.001
Diabetes mellitus	1.430 (1.056–0.935)	0.021		
Active malignancy	2.106 (1.535–2.889)	<0.001	2.447 (1.728–3.465)	<0.001
Modified CCI***	1.064 (1.012–1.118)	0.016		
Sepsis	1.298 (0.952–1.771)	0.099		
Unknown cause	0.173 (0.023–1.329)	0.092		
Hemoglobin	0.882 (0.829–0.939)	<0.001		
Platelet	0.997 (0.996–0.998)	<0.001	0.998 (0.996–0.999)	<0.001
INR	1.198 (1.052–1.364)	0.007	1.261 (1.089–1.459)	0.002
D-dimer	1.012 (1.004–1.020)	0.004	1.010 (1.003–1.018)	0.007
Albumin	0.684 (0.538–0.873)	0.002		
BUN	1.006 (1.002–1.010)	0.005	1.007 (1.002–1.011)	0.002
Total bilirubin	1.039 (1.009–1.070)	0.011		
Left-sided pleural effusion	1.351 (0.977–1.868)	0.069		
Peri-splenic ascites	1.581 (1.171–2.133)	0.003	1.634 (1.201–2.225)	0.002
Multiple splenic infarction	1.579 (1.116–2.234)	0.010	1.826 (1.253–2.661)	0.002
Total splenic infarction	2.485 (1.244–4.961)	0.010	2.333 (1.092–4.987)	0.029
Main portal vein thrombosis	2.110 (1.260–3.534)	0.005		
Splenic vein thrombosis	1.545 (1.071–2.230)	0.020		
Anticoagulant therapy	0.657 (0.448–0.963)	0.031		
Admission-to-CT Interval	0.998 (0.993–1.002)	0.302	0.993 (0.988–0.999)	0.033

* Variables with p < 0.05 in univariate analyses were entered into multivariate analysis using backward elimination after

adjustment for the admission-to-CT Interval.

**HR denotes the average hazard ratio (AHR) estimated using weighted Cox regression that accommodates potential non-proportional hazards.

***Because malignancy is included in the CCI, a modified CCI was calculated by excluding malignancy-related components to avoid conceptual overlap and potential collinearity with the active malignancy variable in the model.

CI: confidence interval; INR: international normalized ratio; BUN: blood urea nitrogen; CT: computed tomography

Table S8. Sensitivity Analysis of Complete-Case Cox Regression Models for Factors Associated with 1-Year Mortality in Hospitalized Patients with Splenic Infarction

Predictive Variables	Univariate analysis		Multivariate analysis*	
	HR** (95% CI)	*p* value	HR (95% CI)	*p* value
Age	1.012 (1.003–1.021)	0.010		
Diabetes mellitus	1.402 (1.040–1.890)	0.027		
Active malignancy	2.064 (1.520–2.802)	<0.001	2.316 (1.661–3.229)	<0.001
Modified CCI**	1.057 (1.003–1.113)	0.037		
Sepsis	1.496 (1.076–2.081)	0.017		
Unknown cause	0.129 (0.018–0.920)	0.041		
Hemoglobin	0.864 (0.813–0.919)	<0.001	0.920 (0.860–0.984)	0.016
Platelet	0.997 (0.995–0.998)	<0.001	0.998 (0.996–0.999)	<0.001
INR	1.214 (1.085–1.359)	<0.001		
D-dimer	1.010 (1.003–1.017)	0.005		
Albumin	0.669 (0.523–0.855)	0.001		
BUN	1.007 (1.002–1.011)	0.004	1.007 (1.003–1.012)	0.002
Total bilirubin	1.047 (1.022–1.071)	<0.001		
Left-sided pleural effusion	1.515 (1.107–2.074)	0.010		
Peri-splenic ascites	1.608 (1.197–2.161)	0.002	1.585 (1.168–2.151)	0.003
Multiple splenic infarction	1.517 (1.080–2.131)	0.016		
Total splenic infarction	2.608 (1.349–5.040)	0.004		
Main portal vein thrombosis	1.870 (1.147–3.049)	0.012		
Splenic vein thrombosis	1.522 (1.009–2.295)	0.045		
Anticoagulant therapy	0.685 (0.471–0.996)	0.047		

* Variables with $p < 0.05$ in univariate analyses were entered into multivariate analysis using backward elimination.

**Because malignancy is included in the CCI, a modified CCI was calculated by excluding malignancy-related components to avoid conceptual overlap and potential collinearity with the active malignancy variable in the model.

CI: confidence interval; INR: international normalized ratio; BUN: blood urea nitrogen

Table S9. Association Between Anticoagulant Therapy and 1-Year Mortality Using Different Analytical Approaches

Model	Method Description	HR (95% CI)	*p* value
Complete-case Cox	Unadjusted complete-case Cox regression	0.685 (0.471–0.996)	0.047
Weighted Cox	Unadjusted weighted Cox regression using hospital admission as the time origin	0.657 (0.448–0.963)	0.031
Weighted Cox	Unadjusted weighted Cox regression using CT diagnosis date as the time origin	0.747 (0.504–1.108)	0.147
IPTW Cox	Propensity score–weighted Cox regression*	1.143 (0.758 –1.720)	0.524

*The propensity score model incorporated clinically relevant baseline covariates associated with both treatment allocation and outcomes. These covariates included age; modified Charlson Comorbidity Index; active malignancy; cardiovascular comorbidities (cerebrovascular accident, transient ischemic attack, coronary artery disease, valvular heart disease, prior myocardial infarction, and congestive heart failure); prior anticoagulant use; laboratory parameters (INR, APTT, hemoglobin, platelet count, and D-dimer); co-infarction; and imaging findings (peri-splenic ascites, portal vein thrombosis, and splenic vein thrombosis).
IPTW: inverse probability of treatment weighting

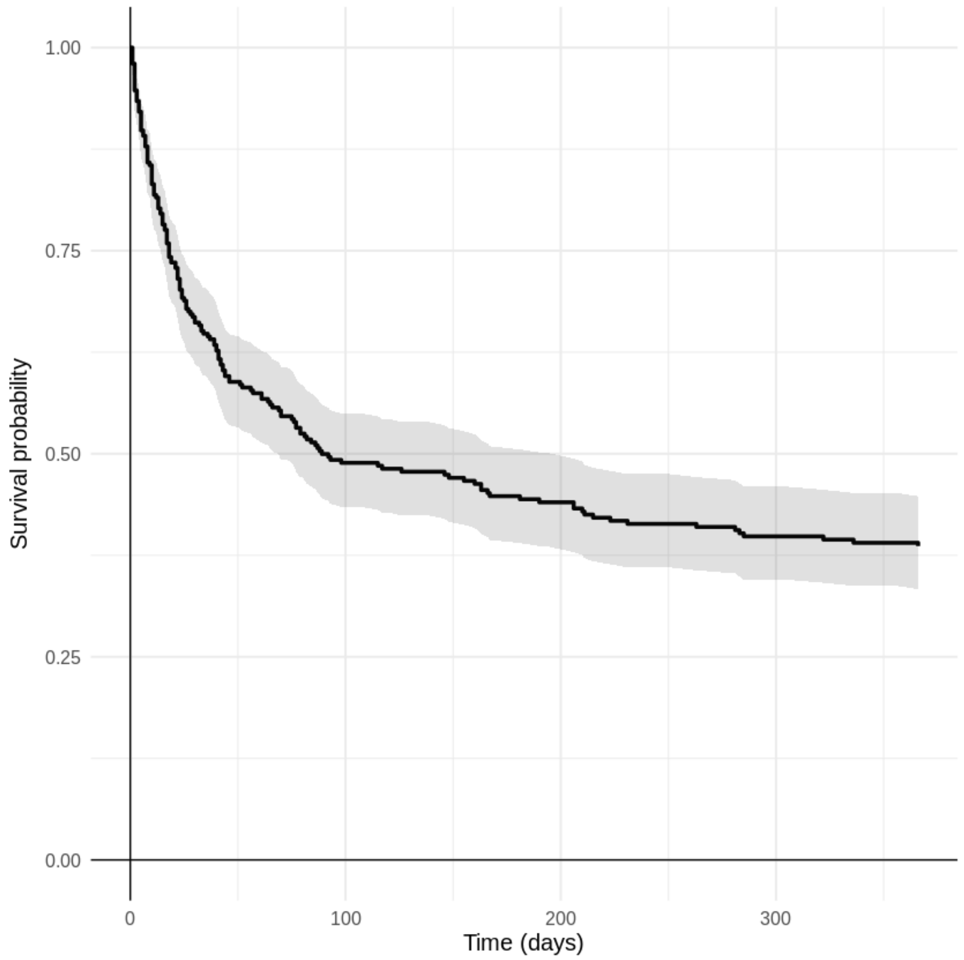

Figure S1. Kaplan–Meier curve in patients with splenic infarction
